# Congenital bronchial atresia presenting as a cavitary lesion on chest radiography: a case report

**DOI:** 10.1186/1757-1626-2-17

**Published:** 2009-01-07

**Authors:** Kostas Psathakis, Danai Eleftheriou, Panagiotis Boulas, Charalampos Mermigkis, Kostas Tsintiris

**Affiliations:** 1 Department of Pneumonology, Army General Hospital of Athens, Athens, Greece

## Abstract

**Background:**

Congenital bronchial atresia is a rare anomaly, which usually presents in adulthood as an incidental finding on routine examinations.

**Case presentation:**

In this report we present a patient with a cavitary lesion at his right upper lobe, found by chance on chest radiography. Computed tomography of the chest revealed the characteristic findings of a mucocele with distal oligemia and hyperlucency of the affected lung parenchyma. Further examination including bronchoscopy virtually excluded other possible disorders and the diagnosis of congenital bronchial atresia was established.

**Conclusion:**

The radiological presentation of congenital bronchial atresia may occasionally mimic serious lung diseases. The procedure of choice for the diagnosis is the computed tomography of the chest. Bronchoscopy is not diagnostic but is valuable in doubtful cases to exclude different disorders.

## Background

Bronchial atresia is a congenital anomaly where the lumen of a bronchus is interrupted at or near its origin. The mucus-filled, blind-terminating bronchus gives a variety of radiographic images in otherwise healthy individuals.

## Case presentation

A 26-year-old male was admitted to our hospital because of an abnormal chest x-ray that was performed in the context of a routine examination.

The chest x-ray revealed a cavitary lesion at the right upper lobe. The patient was a non-smoker, had no symptoms and denied any chronic disease. He only mentioned three incidents of pneumonia at the ages of 3, 12 and 20. The physical examination was normal. The routine laboratory tests were also within normal limits. PPD skin test was negative. Further work-up of the patient, including serum complement analysis, rheumatoid factor, antinuclear antibodies, antineutrophilic cytoplasmic antibodies, immunoglobulin levels, serologic tests for human immunodeficiency virus as well as for common viruses and atypical infectious agents, did not reveal any abnormal findings.

**Figure 1:**
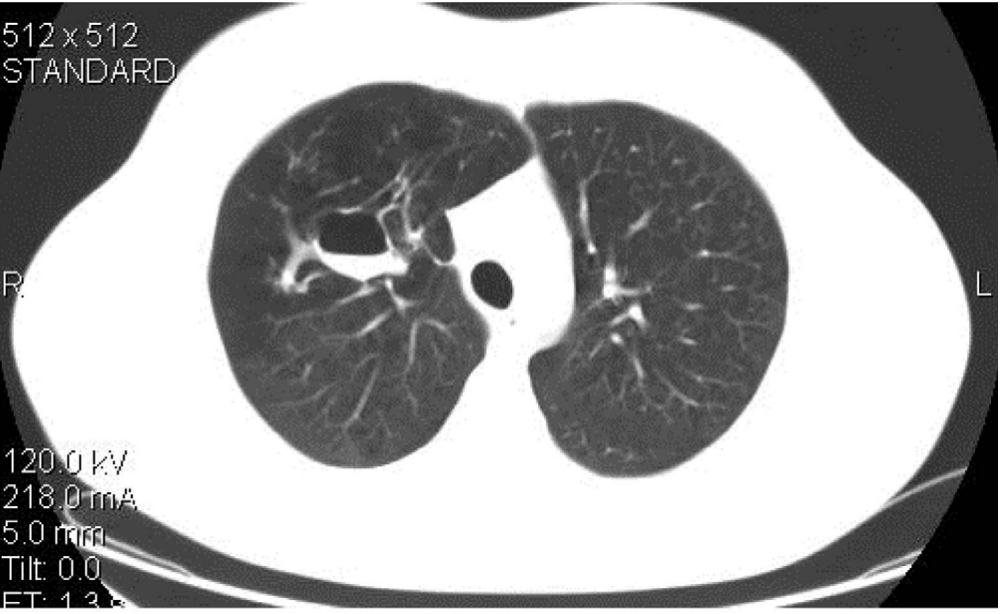
**A CT scan of the chest reveals the cavitary lesion with the air-fluid level in it and the distal oligemia and hyperlucency of the lung**.

A computed tomography (CT) scan of the chest was performed, which revealed a thin-wall cavity at the right upper lobe with the presence of an air fluid level in it. The lesion was surrounded by an area of hyperlucent lung parenchyma and communicated with a segmental bronchus (Figure 1). These findings were consistent with a mucocele.

**Figure 2:**
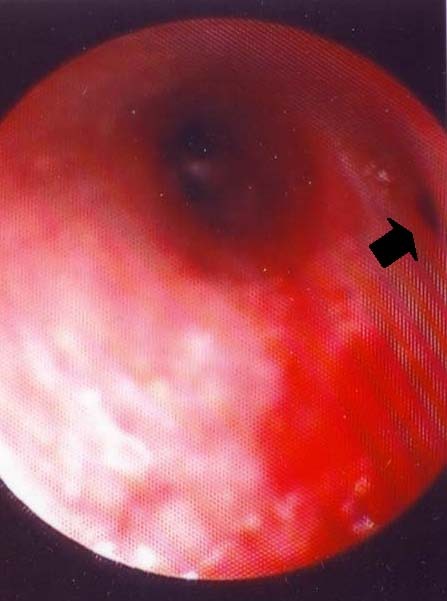
**Bronchoscopic image: the two subsegmental bronchi of the anterior bronchus of the right upper lobe can be seen**. A small orifice at the upper wall of the anterior segmental bronchus is visible (arrow), which might correspond to the atretic apical segment.

Bronchoscopy revealed two segmental bronchi for the right upper lobe that corresponded to the posterior and anterior segments respectively. A very small orifice at the beginning of the anterior segmental bronchus perhaps corresponded to the "atretic" apical segment (Figure 2). Microbiologic and cytologic examinations of the washing specimens from the area were negative.

The patient discharged with the diagnosis of congenital bronchial atresia without further intervention. On follow-up, three years later, he remained asymptomatic without any changes on the CT scan of the chest.

## Discussion

Bronchial atresia is characterized by a mucocele (or bronchocele) resulting from a mucus-filled, blind-terminating sub-segmental, segmental or lobar bronchus, at or near its origin, and hyperinflation of the isolated lung parenchyma. More than 100 cases have been reported in the English literature since 1953, when the abnormality was first described [1].

The lung parenchyma that is to be supplied by the affected bronchus is usually emphysematous, non-compressible, non-inflamed, and minimally anthracotic because it does not communicate directly with the environment. The air that exists in the affected parenchyma is the result of the collateral ventilation through the pores of Kohn, the bronchoalveolar channels of Lambert or via interbronchiolar channels. The process of hyperinflation may occur shortly after birth with the start of respiration, since the proposed pathways for collateral ventilation favour the movement of air into the obstructed segment by a check-valve type mechanism. At the root of the involved tissue a mucus filled cystic structure (the mucocele) with finger-like projections represents the atretic bronchus, which is isolated from the proximal bronchial tree and is dilated by the accumulated mucus. The bronchial pattern distally to the mucocele is usually normal [2,3].

Bronchial atresia is usually diagnosed in the second or third decade of life. It seems that the disorder has a male predominance, with an estimated prevalence of 1.2 cases per 100,000 males [4]. The insidious course of the disorder explains its late detection in some patients. About half to two thirds of the reported patients had been asymptomatic before diagnosis. Recurrent pneumonias, dyspnea, cough or haemoptysis have been reported less frequently [3,4].

On chest radiograph the typical findings of a mucocele is that of a nodule or a mass like shadow close to the hilum, with well-defined margins, which usually forms the apex of a roughly triangular zone of hyperlucency of the lung parenchyma (due to oligemia and hyperinflation). However, the synchronous appearance of both the mucocele, as described above, and the lung hyperlucency, on the same radiograph, is not always seen (69%) [3].

CT remains the procedure of choice for the diagnosis and study of congenital bronchial atresia. Chest CT, especially using high resolution technique, can display exquisitely the characteristic features of the mucocele and is more sensitive, than the conventional chest radiograph, for the demonstration of the oligemia, the reduced size of the pulmonary vessels and the hyperinflation of the lung parenchyma [5,6].

Bronchoscopy may identify a blind-ending bronchus, but it may be normal as well. In clinical practice however, any absence of a segmental or sub-segmental bronchus that is found by chance during bronchoscopy, in the absence of the characteristic radiographic features, may be considered as a normal anatomic variance of the bronchial tree rather than a bronchial atresia. From this point of view, in the majority of cases, congenital bronchial atresia remains a radiological diagnosis. The CT findings (mucocele with hyperaeration of the adjacent lung parenchyma) are considered pathognomonic by most authors [6]. However, some publications suggest that similar findings could be found in serious disorders as well, such as lung cancer or bronchial adenoma [7,8]. The role of bronchoscopy is to exclude these disorders and demonstrate the patency of the central bronchi, especially in doubtful cases [5,9].

A mucocele with an air-fluid level is considered as a variance of the radiographic images of congenital bronchial atresia [3,6]. Some authors believe that this finding is encountered in case of an infection [2], and this is consistent with the presented patient who had a history of pneumonias. Although the exact mechanism of this finding remains obscure (given the overall rarity and benign course of this disorder), it has been proposed that with infection at the area the impacted mucous may liquefy producing the radiological sign of an air-fluid level [3]. In fact the described "cavitary lesion" was not a cavity that was produced after a destructive pneumonia, but a distended (by the mucous) airway, which after the liquefaction of the impacted mucous, as a result of an infection, took the appearance of a "cavity" with an air-fluid level in it.

We conclude that congenital bronchial atresia is a rare and benign entity, which might occasionally resemble serious underlying diseases on radiographic examination. The CT scan of the chest is the procedure of choice for the diagnosis, but in doubtful cases bronchoscopy may be useful to exclude other conditions.

## Competing interests

The authors declare that they have no competing interests.

## Authors' contributions

KP wrote the manuscript. DE had the responsibility of the patient during his hospital stay and the patient's data collection. PB had the responsibility of the bibliographic review and references. CM reviewed the manuscript and processed the photos. KT had the overall responsibility of the patient management and the final approval of the manuscript.

## Consent

Written informed consent was obtained from the patient for publication of this case report and accompanying images. A copy of the written consent is available for review by the Editor-in-Chief of this journal
